# Correction: Equid Herpesvirus Type 1 Activates Platelets

**DOI:** 10.1371/journal.pone.0237679

**Published:** 2020-08-19

**Authors:** Tracy Stokol, Wee Ming Yeo, Deborah Burnett, Nicole DeAngelis, Teng Huang, Nikolaus Osterrieder, James Catalfamo

Due to an error in the gating strategy used for the flow cytometry experiments, the data reported in a number of figures in this article [1] are incorrect, and the conclusion that Equid Herpesvirus Type 1 induced vesiculation independently of thrombin generation is not fully supported.

In experiments conducted after completion of this study, the authors discovered that the virus (presumably in aggregates with protein) was detected by the flow cytometer and also bound Annexin V, the marker for phosphatidylserine expression; as such, the gating strategy used to define platelet-derived microparticles (i.e. low forward scatter, double positive CD41 and Annexin V events) included viral particles. The data was thus re-analyzed (after publication) to reduce the effect of free virus on results. An additional control sample containing only virus was used to isolate and exclude these events in the analysis. This resulted in small changes in the percentage of platelets expressing P-selectin (usually increased) and percentages of platelet-derived microparticles (PDMP, usually decreased), as defined by the new gating strategy. The figures were accordingly revised (and now include individual data points) and the statistical analysis was redone. The significance of some comparisons changed with the revision (see below for detail). However, importantly, the pattern of changes for all the data remain the same. The discussion of results is altered to reflect any changes in statistical significance and to remove one conclusion that there was a thrombin- and tissue factor (TF)-independent component to the vesiculation, as this is no longer supported by the revised data. The remaining conclusions remain unchanged, which are:

- The RacL11 and Ab4 strains of EHV-1 induce platelet activation *ex vivo*, characterized by α-granule exteriorization with P-selectin expression and microvesiculation
- EHV-1-induced platelet activation is mediated by tissue factor on the virus triggering thrombin generation in a plasma milieu and is dependent on factor VII and factor X.
- EHV-1-induced platelet activation is not mediated by glycoprotein C (gC) on the virus, as shown by an Ab4 deletion mutant of gC.
- Retrieval of virus from washed platelets suggests a direct virus-platelet interaction

Here we provide revised Figs 2, 3, 4, 5, 6 and 8 and their revised captions using the results of the new analyses.

**Fig 2:**
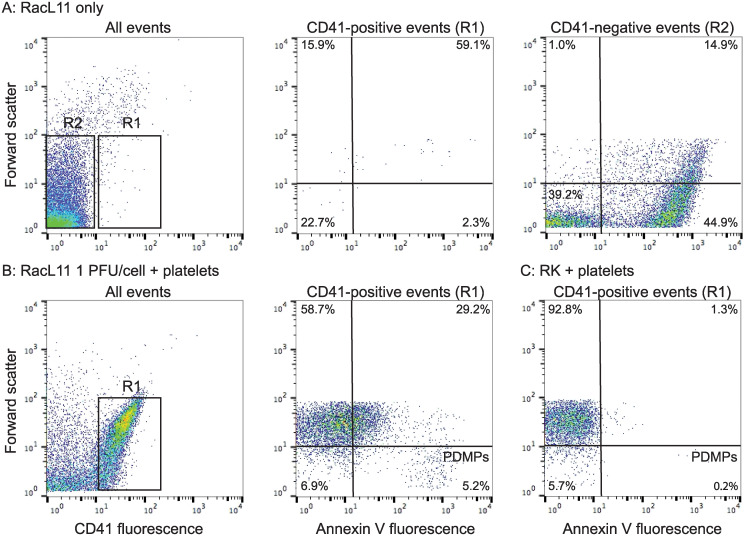
Revised flow cytometry gating strategy for quantification of platelet-derived microparticles in equine platelet samples. **A**: Non-virus events in samples containing virus only (RacL11 shown) were identified and gated as CD41-positive events (R1 region) in a CD41 fluorescence versus forward scatter dotplot. Virus events were defined as CD41-negative events (R2, left panel). Large numbers of events (>100,000) were counted in the virus alone sample to optimize establishment of the gate. Quadrant regions of an Annexin V fluorescence versus forward scatter plot showed few Annexin V-positive events (middle panel, R1 gate), whereas many (57%) of the CD41-negative virus events were positive for Annexin V, with about 80% being small events (<101 log forward scatter units, right panel, R2 gate). **B**: Representative images of platelets in citrated platelet-rich plasma exposed to the RacL11 strain of EHV-1 at 1 plaque forming unit (PFU)/cell. Platelet-derived microparticles (PDMPs) were defined as small events (<101 log forward scatter units) that were double positive for CD41 and Annexin V. CD41-positive events were first defined as above (R1 gate, left panel), then the PDMP percentage was obtained from the lower right quadrant of an Annexin V fluorescence dotplot of the R1 gate, with positive fluorescence for Annexin V being defined on a sample with no added Annexin V. In this sample, there are 8.7% PDMPs. The events in the upper left and right quadrants are platelets that are negative (58%) and positive (25%) for Annexin V, respectively. Note, that the Annexin V-positive platelet events (upper right quadrant) could reflect virus bound to platelets (since virus alone binds Annexin V) versus phosphatidylserine exteriorization on the platelet surface. **C**: Representative image of PDMP quantification in platelets exposed to rabbit kidney (RK) cell lysate at an equivalent volume to 1 PFU/cell (mock-infected control). In this sample, there are 0.1% PDMPs (lower right quadrant) and 1% of platelets are weakly positive for Annexin V (upper right quadrant).

**Fig 3:**
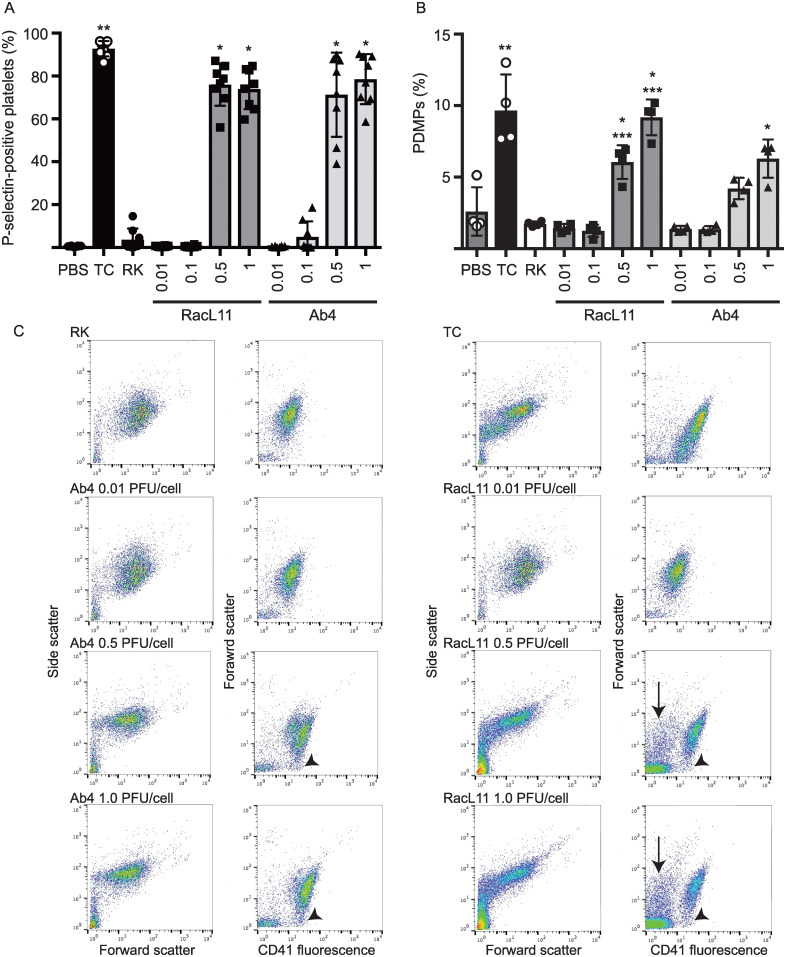
EHV-1 induces platelet P-selectin expression and shedding of platelet-derived microparticles in equine platelet-rich plasma. Platelets were exposed for 10 minutes to RacL11 or Ab4 EHV-1 strains at increasing PFU/cell (0.01, 0.1, 0.5, or 1) with PBS and RK lysate as negative controls and thrombin-convulxin (TC, 0.15 U/mL-0.05 ug/mL) as a positive control. Then the mean percentage ± SD of platelets positive for P-selectin (**A**, n = 8) and PDMPs (**B**, n = 4) were quantified. At the higher PFU/cell of 0.5 and 1, both strains induced P-selectin expression and microvesiculation. Dotplots of forward versus side scatter (**C**, left panels) and CD41 fluorescence versus forward scatter (**C**, right panels) for RK lysate, TC and both viruses show that, at the higher PFU/cell of 0.5 and 1, both strains caused compaction and narrowing of the platelet event cloud and increased CD41-positive small events (arrowheads). At these higher PFUs/cell, the RacL11 strain caused more vesiculation (note fewer events in platelet cloud), with more CD41-negative smaller events (arrows) than the Ab4 strain at equivalent PFUs/cell. CD41-negative events include virus aggregates, which are difficult to distinguish from small platelet events on the forward versus side scatter plot. Exposure to both viruses at 5 PFU/cell replicated these findings, demonstrating dose-dependent microvesiculation (S1 Fig). * p ≤ 0.001 versus PBS or RK negative controls for each virus strain, ** p < 0.001 versus both viral strains at higher PFU/cell (0.5, 1) for P-selectin and p < 0.001 versus Ab4 at the higher PFU/cell (0.5, 1) and versus RacL11 at 0.5 PFU/cell for PDMPs, *** p < 0.001 versus Ab4 at the higher PFU/cell (0.5, 1).

**Fig 4:**
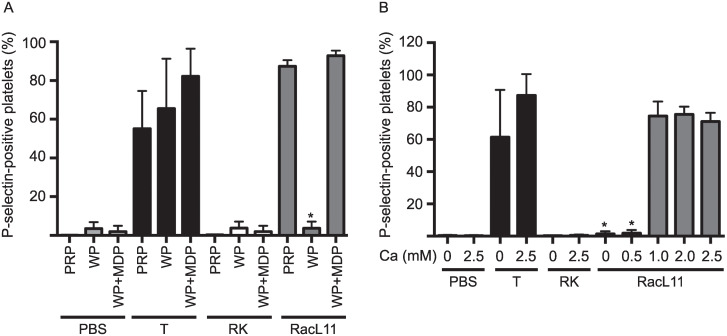
10 minutes to RacL11 at 1 PFU/cell with PBS and RK lysate negative controls and a thrombin (T, 0.15 U/mL) positive control. Washed platelets did not express P-selectin when exposed to virus unless plasma was present. In contrast, thrombin-induced P-selectin expression was independent of plasma (n = 5). Data represents mean ± SD. * p < 0.001 versus PRP or WP + MDP for RacL11-exposed platelets. **B**: Effect of calcium: Equine citrate-anticoagulated PRP was exposed to RacL11 at 1 PFU/cell for 10 minutes with increasing calcium concentrations (0 to 2.5 mM), with the above controls. EHV-1-induced P-selectin expression required at least 1 mM of exogenous calcium (n = 3). Data represents mean ± SD. * p < 0.001 versus 1.0, 2.0 or 2.5 mM calcium.

**Fig 5:**
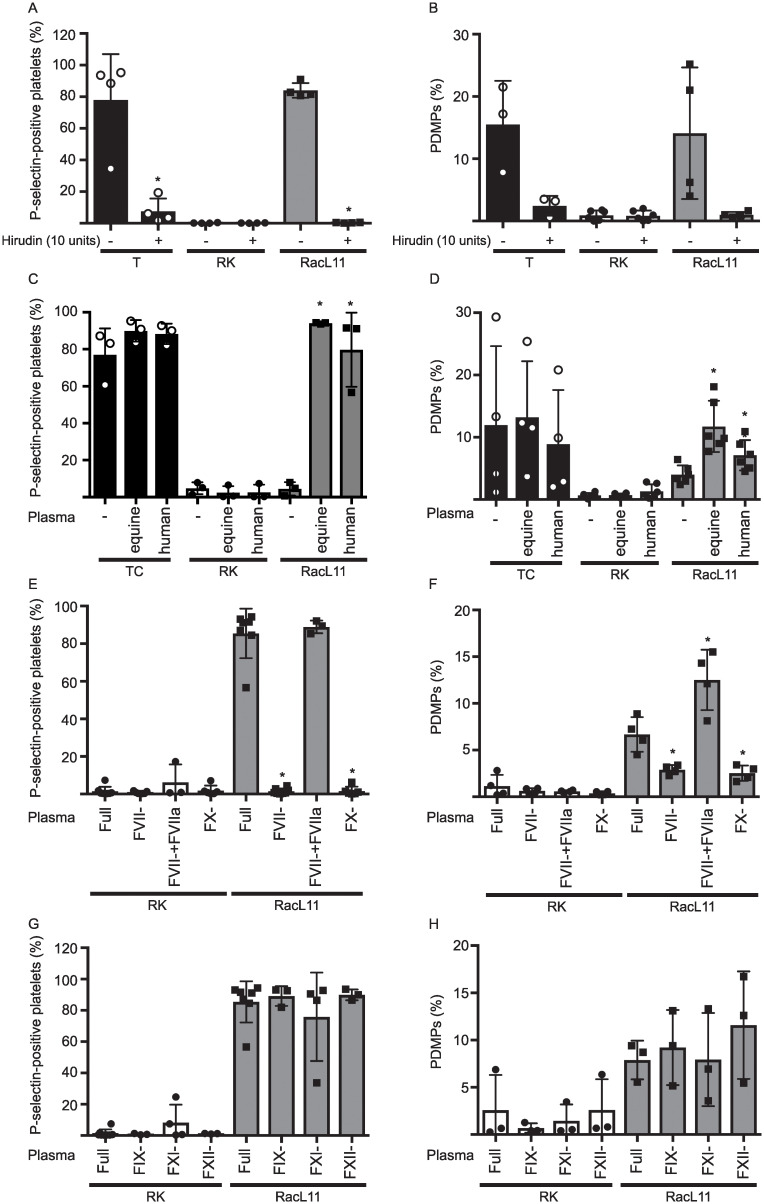
11-exposed washed platelets. However, supplementation of FVII-deficient MDP with purified human FVIIa (1 nM, FVII- + FVIIa) re-established P-selectin expression induced by RacL11, indicating FX generation was secondary to extrinsic pathway activation (**E**, n = 3 to 7). Microvesiculation was also significantly reduced in FVII- and FX-deficient MDP, with supplemental FVIIa boosting the response in FVII-deficient MDP (**F**, n = 4). * p <0.05 versus Full MDP. In contrast, addition of human FIX-, FXI- or FXII-deficient MDP to washed platelets did not significantly affect P selectin expression (**G**) or PDMP release (**H**) (n = 3 to 7). Similar results were seen with Ab4 (S2 Fig).

**Fig 6:**
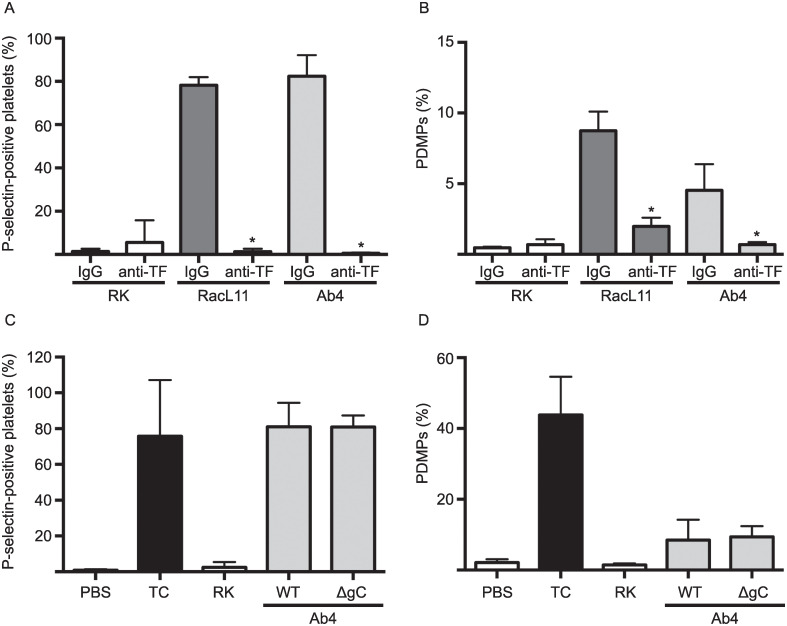
Tissue factor mediates EHV-1-induced platelet activation, with no role for virus glycoprotein C. A goat polyclonal anti-rabbit tissue factor (TF) antibody (anti-TF, 152 ug/mL) abolished P-selectin expression (**A**) and significantly decreased the numbers of platelet-derived microparticles (PDMP) (**B**) in equine platelets exposed to RacL11 and Ab4 EHV-1 strains at 1 PFU/cell (n = 6). Data shown is mean ± SD. * p < 0.001 for P-selectin and p = 0.025 for PDMPs versus goat IgG controls. In contrast, platelets still expressed P-selectin (**C**) and shed PDMPs (**D**) when exposed to an Ab4-based envelope glycoprotein C deletion mutant (ΔgC) at 1 PFU/cell (n = 8). Data shown is mean ± SD.

**Fig 8:**
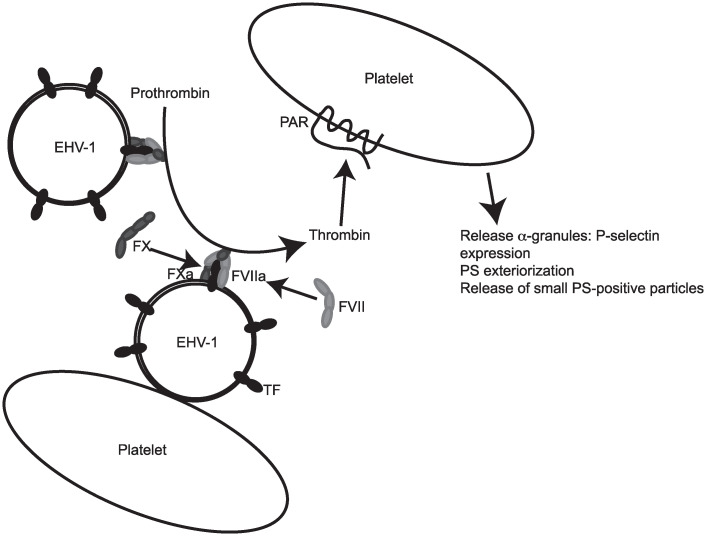
Model for EHV-1-induced activation of platelets. In our model of EHV-1-induced platelet activation, we propose that the virus first binds to platelets. Concurrently, factor VII (FVII) forms a complex with TF in the viral envelope and activates factor X (FX), with FXa generating thrombin in the vicinity of or on the surface of platelets. Thrombin generation may be propagated by phosphatidylserine (PS) in the viral envelope. Thrombin binds to protease-activated receptors (PAR) on platelet surfaces, inducing release of α-granules with subsequent surface P-selectin and PS expression and microvesiculation. If marked, microvesiculation results in loss of membrane-anchored P-selectin on microparticles. Depending on the viral strain or load, thrombin may be generated on the surface of virus not attached to platelets.

Here we provide revised S1, S2 and S3 Figs, and their revised captions using the results of the new analyses.

The complete underlying dataset, including flow cytometric files generated by a BD FACSCAN flow cytometer, jpg files of the analyzed FACS data generated in FlowJo software, version 10, and a Prism file (PZF; GraphPad Prism for Mac, Version 7.0) used to generate the figures and statistically analyze the data, is deposited at Cornell University eCommons Repository (https://doi.org/10.7298/hrds-5r39).

A number of corrections are required to the original article text to adjust specific descriptions of results and to remove conclusions that there was a thrombin- and TF-independent component to the microvesiculation, A revised version of the article text is provided here as Supporting Information (S1 File). In the revised manuscript, additions or changes to original wording are highlighted in yellow and deletions are marked with an inserted comment. Please see the location of corrections required in the original published article and a brief summary of the revisions made here.

**Table:** 

**Location**	**Revision**
Abstract, penultimate sentence	Sentence removed: “Microvesiculation was only partly tissue factor and thrombin-dependent, suggesting the virus causes microvesiculation through other mechanisms, potentially through direct binding.”
Introduction, third paragraph, last sentence	Sentence removed: “In contrast platelet microvesiculation was partly TF- and thrombin-independent”
Materials and Methods, “Platelet activation measurements” subsection, sixth sentence	Two sentences added after the sixth sentence: “In experiments conducted after completion of this study, we discovered that the virus (presumably in aggregates with protein from lysed cells) was detected by the flow cytometer. The data was thus re-analyzed (after publication) to reduce the effect of free virus on results.”
Materials and Methods, “Platelet activation measurements” subsection, seventh sentence	Three sentences added after the seventh sentence: “The gate was set to largely exclude free virus, which is negative for P-selectin and could falsely decrease the percentage of platelets positive for P-selectin. We also found that many of the virus events were positive for Annexin V, but negative for CD41. Since virus alone could falsely increase the PDMP percentage, the data for PDMPs were re-analyzed by defining CD41-positive events on a sample containing virus alone in a CD41 fluorescence versus FSC dotplot.”
Results, “EHV-1 strains, RacL11 and Ab4, activate platelets in a concentration-dependent manner” subsection, fourth-fifth sentence	Removed text making comparisons to thromboxin-convulxin control, which are not supported by the results from the re-gated analyses. The correct text is: “Microvesiculation was more pronounced following exposure to the RacL11 strain, with RacL11 generating significantly more PDMP at higher infectious doses than Ab4.”
Results, “Virus-induced P-selectin expression, and to a lesser extent microvesiculation, requires factor VII, factor X and thrombin” subheading	Section heading revised to “Virus-induced platelet activation requires factor VII, factor X and thrombin”
Results, “Virus-induced P-selectin expression, and to a lesser extent microvesiculation, requires factor VII, factor X and thrombin” subsection, first paragraph, penultimate sentence	Removed text indicating microvesiculation decrease with Hirudin was significant. The correct sentence is: “Hirudin also decreased microvesiculation in RacL11-exposed platelets (Fig 5B).”
Results, “Virus-induced P-selectin expression, and to a lesser extent microvesiculation, requires factor VII, factor X and thrombin” subsection, second paragraph, third sentence	Removed text indicating lower response with human MPD was significant. The correct sentence is: “Unlike P selectin expression, microvesiculation was still present in RacL11-exposed ACD-washed platelets but was enhanced by addition of equine or human MDP, with a lower response being observed with human MPD (Fig 5D).”
Results, “Virus-induced P-selectin expression, and to a lesser extent microvesiculation, requires factor VII, factor X and thrombin” subsection, second paragraph, penultimate sentence	Sentence removed: “Persistence of microvesiculation in plasma-free washed platelets or washed platelets in FVII- and FX-deficient MDP (a similar PDMP percentage was obtained for these three samples) after exposure to virus indicates that microvesiculation is uncoupled from α-granule secretion and that the virus can induce a small degree of microvesiculation in a plasma- and thrombin-independent manner”.
Results, “EHV-1-induced platelet activation is mediated by the tissue factor-factor VIIa complex and not viral glycoprotein C” subsection, first paragraph, first sentence	Sentence added after first sentence: “We also found that virus alone binds Annexin V, suggesting the envelope expresses phosphatidylserine (Fig 2).”
Results, “EHV-1-induced platelet activation is mediated by the tissue factor-factor VIIa complex and not viral glycoprotein C” subsection, second paragraph, second sentence	Updated text to reflect significant inhibition of microvesiculation with anti-TF antibody. The correct sentence is: “The anti-TF antibody abolished P-selectin expression on EHV-1-exposed platelets (Fig 6A) and significantly inhibited microvesiculation (Fig 6B).”
Discussion, first paragraph, second sentence	Removed text indicating a thrombin-independent component to vesiculation. The correct sentence is: “Platelet activation is largely mediated by thrombin generated through TF-FVIIa-triggered activation of FX.”
Discussion, first paragraph, last sentence	Removed text indicating microvesiculation being TF- and thrombin-independent. The correct sentence is: “Direct virus binding to platelets could potentially explain the concentration-dependent nature of the microvesiculation response, which was also partly TF and thrombin-independent.”
Discussion, third paragraph, first sentence	Removed text indicating thrombin generated fewer PDMPs compared to higher infectious viral doses of RacL11 strain. The correct sentence is: “Higher infectious viral doses, particularly with the RacL11 strain, yielded strong microvesiculation.”
Discussion, third paragraph, sixth-seventh sentences	Sentences removed: “Also, microvesiculation was partly thrombin-independent, leading us to speculate that the virus may activate signaling responses or induce changes in the cytoskeleton leading to membrane blebbing. This could be a direct consequence of virus binding to the platelet surface, particularly since the virus does not require plasma or calcium to associate with platelets.”
Discussion, fourth paragraph, sixth-seventh sentences	Sentences revised to reflect findings from re-gated analyses. The correct text is: “It is likely the virus acquires phosphatidylserine from host cell membranes, as described for HSV and human cytomegalovirus [29,39].”

## Supporting information

S1 File Revised article text. (DOCX)

S1 Fig EHV-1 induces platelet activation in equine platelet-rich plasma in a concentration-dependent manner. Platelets were exposed for 10 minutes to RacL11 and Ab4 EHV-1 strains at increasing PFU/cell (0.01, 0.1, 0.5, 1 or 5) with rabbit kidney (RK) lysate as a negative control. The mean ± SD percentage of platelets positive for P-selectin (**A**) or PDMPs (**B**) was then quantified (n = 4). At the higher PFUs/cell of 0.5, 1 and 5, both strains induced P-selectin expression and microvesiculation. RacL11 induced slightly stronger microvesiculation than Ab4, with a decrease in P-selectin expression at the higher PFU/cell of 1 and 5. * p < 0.001 for P-selectin and p = 0.002 for PDMP versus RK, 0.01 and 0.1 PFU/cell for each virus strain (except Ab4 at 0.5 PFU/cell was not significantly different from RK). ** p < 0.001 versus Ab4.
(EPS)

S2 Fig The Ab4 strain of EHV-1 induces platelet activation through factor VII-generated thrombin. Addition of hirudin (10 units) to equine citrate-anticoagulated platelet-rich plasma reduced P-selectin expression (**A**) and release of platelet-derived microparticles (PDMPs; **B**) in response to Ab4 at 1 plaque forming unit/cell or thrombin (T, 1 U/mL) (n = 3–5). No P-selectin expression or PDMP release occurred in PBS-treated negative control platelets. * p < 0.05 versus untreated platelets. P-selectin expression was abolished in washed platelets exposed to Ab4, but re-established with addition of platelet-derived microparticle-depleted citrate-anticoagulated equine (E) or human plasma containing all coagulation factors (Full) or human plasma deficient in factors IX, XI or XII. In contrast, addition of human FVII- or FX-deficient plasma did not re-establish P-selectin expression, unless supplemental purified FVIIa (1 nM) was added to FVII-deficient plasma (FVII- + FVIIa) (**C**, n = 4). * p < 0.05 versus washed platelets with no added plasma, ** p < 0.05 versus Full plasma. The release of PDMPs was boosted in virus-exposed washed platelets in the presence of equine or human plasma. The degree of microvesiculation was significantly decreased when FVII- or FX-deficient plasma was added to Ab4-exposed washed platelets and supplemental purified human FVIIa significantly boosted PDMP percentages in FVII-deficient plasma (**D**, n = 3). Data shown are mean ± SD. * p < 0.05 versus washed platelets with no added plasma, ** p < 0.05 versus Full plasma.
(EPS)

S3 Fig Equid herpesvirus type 1 (EHV-1)-induced platelet activation is not affected by corn trypsin inhibitor (CTI). Platelet-rich plasma prepared from blood collected into citrate anticoagulant with or without CTI (50 ug/mL) was exposed to the RacL11 and Ab4 strains of EHV-1 at 1 plaque forming unit/cell or rabbit kidney (RK) cell lysate for 10 minutes at 37°C, then the mean ± SD percentage of platelets expressing P-selectin (**A**) or platelet-derived microparticles (PDMPs, **B**) was quantified by flow cytometry (n = 3). CTI did not significantly inhibit these markers of platelet activation.
(EPS)
