# Supplementary material for: Correction: Equid Herpesvirus Type 1 Activates Platelets
Source: PLoS One. 2020 Aug 19;15(8):e0237679. doi: 10.1371/journal.pone.0237679 (PMC7446791; doi:10.1371/journal.pone.0237679)
Supplement: S1 File — (DOCX) [file pone.0237679.s001.docx]

Equid herpesvirus type 1 activates platelets

Tracy Stokol^1*^, Wee Ming Yeo^1,#a^, Deborah Burnett^1,#b^, Nicole DeAngelis^1^, Teng Huang^2^, Nikolaus Osterrieder^2^, James Catalfamo^1^

^1^ Department of Population Medicine and Diagnostic Sciences, College of Veterinary Medicine, Cornell University, Ithaca, NY, USA.

^2^ Institut für Virologie, Freie Universität Berlin, Berlin, Germany.

^#a^ Current address: SUTL EnvironTech Pte Ltd, Singapore, Republic of Singapore.

^#b^ Current address: Faculty of Veterinary Science, University of Sydney, Sydney, Australia.

* Corresponding author

Email: tracy.stokol@cornell.edu

Short title: EHV-1-induced platelet activation

**Abstract**

Equid herpesvirus type 1 (EHV-1) causes outbreaks of abortion and neurological disease in horses. One of the main causes of these clinical syndromes is thrombosis in placental and spinal cord vessels, however the mechanism for thrombus formation is unknown. Platelets form part of the thrombus and amplify and propagate thrombin generation. Here, we tested the hypothesis that EHV-1 activates platelets. We found that two EHV-1 strains, RacL11 and Ab4 at 0.5 or higher plaque forming unit/cell, activate platelets within 10 minutes, causing α-granule secretion (surface P-selectin expression) and platelet microvesiculation (increased small events double positive for CD41 and Annexin V). Microvesiculation was more pronounced with the RacL11 strain. Virus-induced P-selectin expression required plasma and 1.0 mM exogenous calcium. P-selectin expression was abolished and microvesiculation was significantly reduced in factor VII- or X-deficient human plasma. Both P-selectin expression and microvesiculation were re-established in factor VII-deficient human plasma with added purified human factor VIIa (1 nM). A glycoprotein C-deficient mutant of the Ab4 strain activated platelets as effectively as non-mutated Ab4. P-selectin expression was abolished and microvesiculation was significantly reduced by preincubation of virus with a goat polyclonal anti-rabbit tissue factor antibody. Infectious virus could be retrieved from washed EHV-1-exposed platelets, suggesting a direct platelet-virus interaction. Our results indicate that EHV-1 activates equine platelets and that α-granule secretion is a consequence of virus-associated tissue factor triggering factor X activation and thrombin generation. These findings suggest that EHV-1-induced platelet activation could contribute to the thrombosis that occurs in clinically infected horses and provides a new mechanism by which viruses activate hemostasis.

**Introduction**

Viruses can activate the hemostatic system, resulting in a hypercoagulable state that may manifest as thrombosis or disseminated intravascular coagulation [1-3]. The mechanisms underlying virus-associated thrombosis are poorly understood, however virus-induced expression of tissue factor (TF) on monocytes and endothelial cells may be involved [3,4]. Platelets also play crucial roles in hemostasis. Activated platelets bind coagulation factor complexes on their phosphatidylserine-bearing membrane surfaces, amplify factor activity, and accelerate fibrin formation [5]. They shed phosphatidylserine-rich membrane microparticles (PDMPs), which are strongly procoagulant [6]. Activated platelets also promote inflammation, recruiting leukocytes through P-selectin-P-selectin glycoprotein ligand-1 (PSGL-1) interactions [7]. Inhibition of P-selectin-PSGL-1 reduces thrombus formation and inflammation in murine models *in vivo* [8,9]. Various viruses bind to and are internalized by platelets [10-14] and platelets are activated during viral infection [15,16]. This data suggests that viruses may activate platelets, thus directly contributing to thrombosis in virus-infected patients.

Equid herpesvirus type 1 (EHV-1) is a double stranded DNA virus and a member of the Alphaherpesviridae subfamily. EHV-1 is highly contagious causing outbreaks of respiratory and neurologic disease, abortion, and neonatal mortality [17]. Similar to human patients with herpes simplex (HSV) and varicella-zoster virus infection [1], thrombi are found in vessels of EHV-1-infected horses [18-20]. Thrombosis-induced ischemic tissue injury likely contributes to the pathogenesis of the clinical syndromes of abortion, neonatal mortality and neurologic disease. The mechanisms of thrombosis with EHV-1 infection are, however, largely unknown. Thrombosis could be secondary to endothelial cell infection by the virus, which results in a leukocytoclastic vasculitis [19-21]. Also, we have recently shown that EHV-1 infection induces tissue factor (TF) expression in monocytes [22], which likely contributes to the formation of thrombi in horses with EHV-1 infection.

The role of platelets in the pathogenesis of EHV-1 infection is unknown. Since platelets are integral to thrombosis, we hypothesized that EHV-1 would associate with and activate equine platelets. We found that platelets were activated within 10 minutes of exposure to two EHV-1 strains, RacL11 and Ab4, and released α-granule contents and underwent microvesiculation. The α-granule secretion was mediated by thrombin, generated by virus-associated TF-triggered activation of factor X (FX). **Materials and methods**

All reagents were from Sigma-Aldrich (St Louis, MO) unless otherwise stated. **Preparation of virus and mock controls**

The RacL11 and Ab4 strains of EHV-1 plaque purified strains (<3 passages) were used in the study. RacL11 was isolated from an aborted fetus [23], whereas Ab4 was isolated from a quadriplegic gelding [18]. To abrogate the expression of the Ab4*ORF16* gene, which encodes envelope glycprotein C, *en passant* mutagenesis was performed using an infectious bacterial artificial chromosome (BAC) clone of Ab4 (pAb4) as previously described [24]. Two primers, delgC_Fw and delgC_Rv (Table 1), containing 60 nucleotides for homologous recombination were designed for recombination. Using a kanamycin resistance gene (*Kan^R^*) as a template, a PCR fragment was amplified, digested with *DpnI* to remove residual template DNA, and then the purified product was electroporated into *E. coli* GS1783 (a gift from Dr. Greg Smith, Northwestern University, Chicago, IL, USA)-competent cells containing pAb4. The first round of Red recombination produced kanamycin resistant (*Kan^R^*) colonies, which were screened for correctness by restriction digest. The *Kan^R^* gene was excised using growth in LB medium that was supplemented with 1% L-(+)-Arabinose (Alfa Aesar, Ward Hill, MA, USA). After a second step of Red recombination, *Kan^R^* -deficient colonies were isolated and analyzed by restriction enzyme digestion, PCR verification, and DNA sequencing (Table 1, Fig. 1). The recombinant virus, termed Ab∆gC, was reconstituted by transfection of the pAb4∆gC BAC DNA into rabbit kidney 13 (RK) cells using polyethylenimine (Polysciences Inc, Warrington, PA, USA).

**Table 1. Synthetic oligonucleotides for engineering and sequencing of the gC deletion mutant of Ab4**

| Primer | Sequence (5' → 3') |
| --- | --- |
| **Mutagenesis** |  |
| delgC_Fw | GCGATATTAACTTATGCCTCTGGAGCTAGTGCTAGCTCCACCGCGGCACTGGCGCTGGTCCCAGTGTTACAACCAATTAACC |
| delgC_Rv | CAGACGGCTGTGATGAGAACGACCAGCGCCAGTGCCGCGGTGGAGCTAGCACTAGCTCCATAGGGATAACAGGGTAATCGATT |
| **Sequencing** |  |
| gC_Fw | CTCCGACCAGTGGAGTTATTAT |
| gC_Rv | CTACTGTTTTTACCAGCGCTTC |

Note: the underlined sequences correspond to the annealing sites of a *Kan^R^* gene.

**Fig. 1: Construction and verification of a glycoprotein C (gC) deletion mutant of Ab4.**

**A:** An illustrative overview of generation of the deletion mutant in an infectious artificial bacterial chromosome (BAC) containing plaque purified Ab4 (Ab4). A PCR product that harbored the *kan_R_* gene instead of nucleotides 98-1315 of the *ORF16* gene, which encodes gC, was electroporated into *E. coli* GS1783 for *en passant* (two-step) Red recombination. Intermediate colonies were screened and used for the second recombination step. Removal of *Kan^R^* was induced by 1% arabinose resulting in the final construct. **B:** Identity of the mutant (Ab4GΔgC) was confirmed by restriction enzyme digests. BAC DNA from the parental, intermediate and final constructs was digested with *HindIII* and separated by 0.8% agarose gel electrophoresis. The intermediate construct harboring *Kan^R^* had an additional band of 6.8 kbp (lower arrow). After resolution, a fragment of 8.6 kbp (upper arrow) containing the *ORF16* gene disappeared and a fragment of 7.4 kbp in the final mutant appeared. This apparent reduction is in line with the deletion introduced by mutagenesis. **C:** BAC DNA from Ab4 (wild-type) or Ab4GΔgC was used as the template for PCR using gC primers that span the deleted region (forward: 5’- CTCCGACCAGTGGAGTTATTAT-3’; reverse: 5’- CTACTGTTTTTACCAGCGCTTC-3’). Bands of the predicted size were amplified in either case, thus confirming deletion of gC-encoding sequences in the mutant Ab4GΔgC.

Virus was propagated in RK cells (provided by Dr. Dubovi, Cornell University) and purified on a discontinuous sucrose gradient using ultracentrifugation. The RK cells were cultured in 75 cm^2^ flasks in Dulbecco’s Modification of Earl’s Medium (Mediatech Inc. Manasses, VA, USA) supplemented with L-glutamine, glucose, sodium pyruvate and 10% fetal bovine serum (Atlanta Biologicals, Inc., Flowery Branch, GA, USA) at 37°C with 5% carbon dioxide in a tissue culture incubator. One-day-old confluent monolayers of RK cells were infected with virus at 0.1 plaque-forming units (PFU)/cell until a cytopathic effect of approximately 90% was achieved. The flasks were then subjected to two freeze-thaw cycles. The culture media were centrifuged at 1800 *x g* for 15 minutes at 4°C then were ultracentrifuged at 175,000 x *g* for 1 hour at 4°C (with no brake) on a 60, 30 and 10% sucrose gradient. The band (containing virus) between the 30% and 60% sucrose layers was removed, diluted in phosphate-buffered saline (PBS, pH 7.4) and ultracentrifuged again. The supernatant was discarded and the pellet was reconstituted in PBS, separated into aliquots and stored at -20°C until use (multiple freeze-thaw cycles were avoided). Virus titers were determined using a standard plaque assay. Freeze-thaw lysates from uninfected RK cells were processed similarly and served as mock-infected negative controls.

**Sample collection**

Blood was collected from the jugular vein of clinically healthy horses using an 18-G needle (Covidien, Mansfield, MA, USA) and 6 ml syringe (Covidien) that was prefilled with 3.2% citrate or acid-citrate dextrose (ACD), maintaining an anticoagulant to blood ratio of 1:9 and 1:5, respectively. The needle was inserted first and blood was allowed to drip through the needle for a few seconds before syringe attachment. Blood was then slowly drawn into the syringe. In select experiments, blood was also collected into citrate vacutainer tubes, with or without added corn trypsin inhibitor (CTI, 50 ug/mL, Hematologic Technologies Inc., Essex Junction, VT). The samples were immediately transported to the laboratory for preparation of platelet-rich plasma (PRP) or washed platelets, which occurred within 15-30 minutes of sample collection. The sample collection protocol was approved by the Institutional Animal Care and Use Committee at Cornell University (#2007-0086), which follows several federal and state guidelines, including the U.S. Department of Agriculture Animal Welfare Act (1966), Regulation (C.F.R., 2009) and the Guide for the Care and Use of Agricultural Animals in Research and Teaching (2010).

**Preparation of platelet-rich plasma and washed platelets**

For preparation of PRP, 3.2% citrate-anticoagulated blood from clinically healthy horses was transferred to a 15 ml polypropylene tube and the red blood cells were allowed to settle using gravity sedimentation for 20 minutes at room temperature. The resulting leukocyte-platelet-rich plasma was removed and centrifuged at 250 x *g* at 21°C for 10 minutes. The supernatant or PRP was removed and a platelet count was measured on the PRP using an automated hematology analyzer (ADVIA 2120, Siemens Healthcare Diagnostics Inc, Tarrytown, NJ, USA). For preparation of washed platelets, ACD-anticoagulated blood was diluted in a 1:1 ratio with platelet buffer I (113 mM NaCl, 4.3 mM K_2_HPO_4_, 4.2 mM Na_2_HPO_4_, 24.4 mM NaH_2_PO_4_, 5.5 mM glucose, pH 6.3), then centrifuged at 450 x *g* at 21°C for 5 minutes to obtain the platelet-rich fraction. This fraction was removed and centrifuged at 1250 x *g* at 21°C for 10 minutes. The platelet pellet was then washed three times with platelet buffer I and gently resuspended in platelet buffer II (10 mM HEPES, 137 mM NaCl, 4 mM KCl, 0.5 mM Na_2_HPO_4_, 0.1% glucose, 0.1% bovine serum albumin [BSA], pH 7.4). A platelet count was measured as above. Platelet-rich plasma or washed platelets were rested for 30 minutes at room temperature before exposure to treatments or virus.

**Platelet treatment or virus exposure**

Platelets were diluted (1 x 10^5^ final concentration) in flow buffer (10 mM HEPES, 140 mM NaCl, pH 7.4) with supplemental glycine-proline-arginine-proline (GPRP, an inhibitor of fibrin polymerization). Calcium chloride (2.5 mM) was added to the buffer for most experiments. The platelets were exposed to virus at various PFUs/cell for 10 minutes at 37°C, using PBS and RK lysate as negative controls. As positive platelet activation controls, platelets were stimulated for 10 minutes at 37°C with bovine thrombin (0.15 U/L; 20-30 nM) for P-selectin expression or a mixture of bovine thrombin and convulxin (0.05 ug/mL, DSM Nutritional Products, Herleen, Netherlands) for PDMP quantification (0.15 U/ml of thrombin alone was a poor stimulator of microvesiculation in equine platelets). In select experiments, platelets in PRP were exposed to the thrombin inhibitor hirudin (10 units), a goat polyclonal anti-rabbit TF antibody (a kind gift from Dr. Pendurthi, University of Texas H.S.C at Tyler) with a goat IgG control (Jackson Immunoresearch Laboratories Inc, West Grove, PA, USA), or an inhibitory murine monoclonal antibody against equine MHC-I [25] (clone CZ3, a kind gift from Dr. Douglas Antzack, Cornell University) with a murine isotype control (AbD Serotec, Raleigh, NC, USA). In other experiments, washed platelets were resuspended in HEPES buffer with added equine plasma or human plasma containing all coagulation factors (factor assay control plasma) or deficient in factors VII, IX, X, XI and XII (George King Biomedical, Overland Park, KS, USA). In some reconstitution experiments with washed platelets, purified activated human FVII (FVIIa, Haematologic Technologies Inc.) was included in the HEPES buffer with human FVII-deficient plasma. Platelet-derived microparticle-depleted plasma (MDP) was generated by centrifugation of human or equine plasma at 2,500 x *g* for 20 minutes at 4°C, then centrifugation twice at 22,000 x *g* for 20 minutes at 4°C. This is an accepted method for obtaining PDMP-depleted plasma [26]. The MDP was warmed to room temperature before adding to washed platelets.

**Platelet activation measurements**

Platelet activation was assessed by quantifying the percentage of platelets expressing P-selectin and shed PDMPs using flow cytometry, as described with some modifications [27]. After a 10 minute exposure to virus or controls, platelets (PRP or washed) were incubated with a phycoerythrin (PE)-conjugated antibody against CD41 (1:10 final concentration, clone P2, Beckman Coulter, Brea, CA, USA), a platelet marker [28], for 10 minutes in the dark at room temperature. The cells were then incubated with either a Dylight-A488-conjugated anti-P-selectin antibody (33.3 ng/mL final concentration, clone Psel.KO.2.7, Novus Biologicals, Littleton, CO, USA) or fluorescein isothiocyanate (FITC)-conjugated Annexin V (1:300 final concentration, TACS Annexin V-FITC Apoptosis Detection kit, Trevigen, Gaithersburg, MD, USA) for 10 minutes in the dark at room temperature. In some experiments, an allophycocyanin (APC)-conjugated anti-P-selectin antibody (33.3 ng/mL final concentration, Novus Biologicals) was added with FITC-Annexin V for 10 minutes. The reaction was quenched with 400 ul of flow buffer. The samples were then analyzed with a flow cytometer (FACSCalibur, BD Biosciences) using log settings for forward (FSC) and side scatter (SSC). In experiments conducted after completion of this study, we discovered that the virus (presumably in aggregates with protein from lysed cells) was detected by the flow cytometer. The data was thus re-analyzed (after publication) to reduce the effect of free virus on results. For measurement of P-selectin, platelets were gated on their characteristic size and complexity on a FSC versus SSC dotplot, then the percentage of platelets positive for P-selectin was quantified using frequency distribution curves (histogram plots) with isotype controls. The gate was set to largely exclude free virus, which is negative for P-selectin and could falsely decrease the percentage of platelets positive for P-selectin. We also found that many of the virus events were positive for Annexin V, but negative for CD41. Since virus alone could falsely increase the PDMP percentage, the data for PDMPs were re-analyzed by defining CD41-positive events on a sample containing virus alone in a CD41 fluorescence versus FSC dotplot. Then, the percentage of PDMP were quantified from the number of Annexin V-positive events that were <10^1^ units in size on an Annexin V fluorescence versus FSC dotplot based on the CD41-positive gate (Fig. 2).

**Fig. 2: Revised flow cytometry gating strategy for quantification of platelet-derived microparticles in equine platelet samples.**

**A:** Non-virus events in samples containing virus only (RacL11 shown) were identified and gated as CD41-positive events (R1 region) in a CD41 fluorescence versus forward scatter dotplot. Virus events were defined as CD41-negative events (R2, left panel). Large numbers of events (>100,000) were counted in the virus alone sample to optimize establishment of the gate. Quadrant regions of an Annexin V fluorescence versus forward scatter plot showed few Annexin V-positive events (middle panel, R1 gate), whereas many (57%) of the CD41-negative virus events were positive for Annexin V, with about 80% being small events (<10^1^ log forward scatter units, right panel, R2 gate).

**B:** Representative images of platelets in citrated platelet-rich plasma exposed to the RacL11 strain of EHV-1 at 1 plaque forming unit (PFU)/cell. Platelet-derived microparticles (PDMPs) were defined as small events (<10^1^ log forward scatter units) that were double positive for CD41 and Annexin V. CD41-positive events were first defined as above (R1 gate, left panel), then the PDMP percentage was obtained from the lower right quadrant of an Annexin V fluorescence dotplot of the R1 gate, with positive fluorescence for Annexin V being defined on a sample with no added Annexin V. In this sample, there are 8.7% PDMPs. The events in the upper left and right quadrants are platelets that are negative (58%) and positive (25%) for Annexin V, respectively. Note, that the Annexin V-positive platelet events (upper right quadrant) could reflect virus bound to platelets (since virus alone binds Annexin V) versus phosphatidylserine exteriorization on the platelet surface.

**C**: Representative image of PDMP quantification in platelets exposed to rabbit kidney (RK) cell lysate at an equivalent volume to 1 PFU/cell (mock-infected control). In this sample, there are 0.1% PDMPs (lower right quadrant) and 1% of platelets are weakly positive for Annexin V (upper right quadrant).

**Factor Xa generation in a one-stage amidolytic assay**

Virus at amounts equivalent to 0.1, 0.5 and 1 PFU/cell or RK lysate at an equivalent amount to 1 PFU/cell was incubated with human FVIIa (1 nM, Haematologic Technologies Inc) and FX (75 nM, Haematologic Technologies Inc.) in HEPES buffer (10 mM HEPES, 137 mM sodium chloride, 5 mM calcium chloride, 4 mM potassium chloride, 10 mM glucose, 0.5% BSA, pH 7.4) for 15 minutes at 37°C. Then a chromogenic substrate (Spectrozyme-FXa, 167 µM, Sekisui Diagnostics, Lexington, MA, USA) was added and the color change was measured kinetically every 15 seconds at an optical density of 405 nm at 37°C with a plate spectrophotometer (Spectromax M3, Molecular Devices, Sunnyvale, CA, USA). The rate of change in optical density (Vmax) was recorded for the first 60 seconds (in the linear portion of the curve) and converted to amount of FXa generated (nM) based on a standard curve created from serial dilutions of human FXa (Haematologic Technologies Inc). The standard curve was linear between FXa concentrations of 0.17 and 5.43 nM. Negative controls included virus without added FVIIa, FX or substrate. Assays were run in duplicate and results were averaged.

**Thrombin generation**

Virus-induced thrombin generation was measured in equine MDP using calibrated automated thrombography and a single lot of a commercially available kit, with calibrators (Technothrombin TGA, Technoclone, Vienna, Austria). Equine citrate-anticoagulated plasma can generate thrombin via contact activation in this system. To inhibit factor XIIa and subsequent contact activation, blood was collected from the jugular vein of a single horse into a CTI-containing citrate vacutainer and CTI-MDP was prepared as described above. Virus or RK lysate (both at amounts equivalent to 1 PFU/cell) were added to CTI-MDP, with or without 1 x 10^5^ platelets as PRP (from the same horse as the CTI plasma), with virus or RK lysate alone, platelets, and CTI-MDP alone as negative controls. After a 10 minute incubation at 37°C, a fluorogenic substrate with calcium was added, then fluorescence intensity was measured at one minute intervals for 120 minutes using excitation and emission wavelengths of 360 and 460 nm, respectively, with a plate spectrofluorometer (Spectromax M3). Thrombograms represent a mean of three experiments. Reported results of lag time (minutes) and total thrombin generated (derived from the area under the curve and expressed as nM*minute) were obtained from the manufacturer’s software (SoftMax Pro 6.2.1).

**Statistical analysis**

Data is expressed as means with standard deviation (SD). Means of 2 groups were compared with a paired T test or student T test, as appropriate. Means of 3 or more groups were compared using an Analysis of Variance (ANOVA) or Repeated ANOVA, as appropriate, with a Tukey’s multiple comparison post-hoc test (Prism 5, GraphPad Software, Inc., La Jolla, CA, USA). A p value (two tailed) was set at < 0.05.

**Results**

**EHV-1 strains, RacL11 and Ab4, activate platelets in a concentration-dependent manner**

Both strains of EHV-1 induced surface P-selectin expression and shedding of PDMPs at 0.5-1 PFU/cell, with little to no activation occurring at lower infectious doses (Fig. 3A-B). We also observed changes consistent with activation in the cluster of platelet events on a FSC versus SSC dotplot at the higher PFUs/cell with both virus strains, including narrowing and compaction of the main platelet cluster and microvesiculation (Fig. 3C). Microvesiculation was more pronounced following exposure to the RacL11 strain, with RacL11 generating significantly more PDMP at higher infectious doses than Ab4. The more marked microvesiculation induced by 1 PFU/cell of RacL11 was associated with reduced P-selectin expression. A similar strong microvesiculation response, with loss of P-selectin expression, occurs in equine platelets exposed to calcium ionophore

[27]. There was variability among different virus preparations in the strength of the reactions, however the strain-dependent differences in microvesiculation were maintained across preparations. We also tested a higher infectious dose of 5 PFU/cell in a subset of 4 horses. Whereas P-selectin expression was maximal at 0.5 PFU/cell, a dose-dependent response was seen with microvesiculation induced by both strains, with a concomitant decrease in P-selectin expression associated with the RacL11 strain (S1 Fig). An infectious dose of 1 PFU/cell was selected for subsequent experiments, unless otherwise stated.

**Fig. 3: EHV-1 induces platelet P-selectin expression and shedding of platelet-derived microparticles in equine platelet-rich plasma.**

Platelets were exposed for 10 minutes to RacL11 or Ab4 EHV-1 strains at increasing PFU/cell (0.01, 0.1, 0.5, or 1) with PBS and RK lysate as negative controls and thrombin-convulxin (TC, 0.15 U/mL-0.05 ug/mL) as a positive control. Then the mean percentage ± SD of platelets positive for P-selectin (**A**, n=8) and PDMPs (**B**, n=4) were quantified.. At the higher PFU/cell of 0.5 and 1, both strains induced P-selectin expression and microvesiculation. Dotplots of forward versus side scatter (**C**, left panels) and CD41 fluorescence versus forward scatter (**C**, right panels) for RK lysate, TC and both viruses show that, at the higher PFU/cell of 0.5 and 1, both strains caused compaction and narrowing of the platelet event cloud and increased CD41-positive small events (arrowheads). At these higher PFUs/cell, the RacL11 strain caused more vesiculation (note fewer events in platelet cloud), with more CD41-negative smaller events (arrows) than the Ab4 strain at equivalent PFUs/cell. CD41-negative events include virus aggregates, which are difficult to distinguish from small platelet events on the forward versus side scatter plot. Exposure to both viruses at 5 PFU/cell replicated these findings, demonstrating dose-dependent microvesiculation (**S1 Fig**).

* p ≤ 0.001 versus PBS or RK negative controls for each virus strain, ** p < 0.001 versus both viral strains at higher PFU/cell (0.5, 1) for P-selectin and p < 0.001 versus Ab4 at the higher PFU/cell (0.5, 1) and versus RacL11 at 0.5 PFU/cell for PDMPs, *** p < 0.001 versus Ab4 at the higher PFU/cell (0.5, 1).

**Virus-induced platelet activation requires plasma and exogenous calcium**

To determine if EHV-1-induced activation of platelets required plasma, we washed ACD-anticoagulated platelets free of plasma and exposed them to RacL11 and positive and negative controls in the presence or absence of equine plasma. Using P-selectin as the marker for platelet activation and thrombin alone as the positive control, we found that expression was abolished in washed platelets and re-established by the addition of 5 ul equine MDP (Fig. 4A). We then determined if EHV-1-induced P-selectin expression required exogenous calcium by excluding calcium from the flow buffer. P-selectin expression in equine PRP in response to EHV-1 was abolished in the absence of calcium and was re-established with 1 mM calcium (Fig. 4B). We did not test for PS exposure since Annexin-V binding requires exogenous calcium. This data suggested that EHV-1-induced platelet activation, specifically P-selectin expression, required the presence of coagulation factors in equine plasma and that virus alone was insufficient for this activation event.

**Fig. 4: EHV-1-induced platelet P-selectin expression requires plasma and exogenous calcium.**

**A**: Effect of plasma: Equine platelets in ACD-anticoagulated platelet-rich plasma (PRP), washed platelets (WP) or washed platelets with added ACD-anticoagulated platelet-derived microparticle-depleted plasma (WP+MDP) were exposed for 10 minutes to RacL11 at 1 PFU/cell with PBS and RK lysate negative controls and a thrombin (T, 0.15 U/mL) positive control. Washed platelets did not express P-selectin when exposed to virus unless plasma was present. In contrast, thrombin-induced P-selectin expression was independent of plasma (n=5). Data represents mean ± SD. * p < 0.001 versus PRP or WP + MDP for RacL11-exposed platelets.
**B**: Effect of calcium: Equine citrate-anticoagulated PRP was exposed to RacL11 at 1 PFU/cell for 10 minutes with increasing calcium concentrations (0 to 2.5 mM), with the above controls. EHV-1-induced P-selectin expression required at least 1 mM of exogenous calcium (n=3). Data represents mean ± SD. * p < 0.001 versus 1.0, 2.0 or 2.5 mM calcium.

**Virus-induced platelet activation requires factor VII, factor X and thrombin**

The prototypical Alphaherpesvirus HSV can initiate thrombin generation through extrinsic or intrinsic/contact pathways [29,30]. We used a combination of chemical inhibitors and factor-deficient human plasma to examine the role of coagulation factors in P-selectin expression and microvesiculation. To inhibit thrombin, we added hirudin (10 units) to equine citrate-anticoagulated PRP. Hirudin blocked P-selectin expression induced by thrombin and RacL11, but had no effect on negative controls (Fig 5A). Hirudin also decreased microvesiculation in RacL11-exposed platelets (Fig 5B). Similar results were seen with Ab4 (S2 Fig).

Since factor-deficient equine plasma is not available, we added factor-deficient human MDP to washed equine platelets to examine the role of the extrinsic pathway in EHV-1-induced platelet activation. Addition of 5 ul of equine or human MDP to washed equine platelets was sufficient to re-establish platelet P-selectin expression after exposure to RacL11 (Fig. 5C). Unlike P selectin expression, microvesiculation was still present in RacL11-exposed ACD-washed platelets but was enhanced by addition of equine or human MDP, with a lower response being observed with human MPD (Fig. 5D). Microvesiculation was unaffected by the addition of equine or human MDP alone without virus (Fig. 5D, RK-treated cells), supporting that the centrifugation process depleted plasma of residual PDMPs. This data also showed that human plasma could be used to examine activation responses with equine platelets. We then found that platelet P-selectin was not expressed and microvesiculation was significantly reduced on washed platelets exposed to RacL11 in human MDP deficient in FVII or FX (Fig. 5E-F). This data indicated that EHV-1-induced α-granule secretion and, to a large extent, microvesiculation was FVII-dependent and the virus was not directly activating FX or prothrombin. Accordingly, RacL11-induced platelet P-selectin expression and microvesiculation was re-established by addition of 1 nM of purified human FVIIa to washed platelets in FVII-deficient MDP (Fig. 5E-F). Similar results were seen with Ab4 (S2 Fig).

Herpes simplex virus can also generate thrombin through the contact and intrinsic pathways [30]. Also, the TF-FVIIa complex can activate FX and generate thrombin through the so-called “alternate” pathway via activating FIX. Thus, we tested the contribution of contact and intrinsic pathway enzymatic factors to EHV-1-induced activation using our washed platelet and factor-deficient human MDP reconstitution system. We found that platelet P-selectin expression or PDMP release still occurred in the absence of FIX, FXI and FXII (Fig. 5G-H for RacL11, S2 Fig for Ab4). Also, the percentage of P-selectin-positive platelets or PDMPs was not significantly reduced in EHV-1-exposed platelets in equine PRP with or without CTI (S3 Fig). This data indicates that EHV-1-induced FXa and thrombin generation is driven by extrinsic pathway factors.

**Fig 5. The RacL11 strain of EHV-1 induces P-selectin exteriorization through the extrinsic pathway of coagulation.**

The mean ± SD percentages of P-selectin-positive platelets and platelet-derived microparticles (PDMPs) were quantified in equine platelets in citrate-anticoagulated PRP with or without hirudin (10 units), after exposure to RacL11 at 1 PFU/cell or rabbit kidney (RK) cell lysate negative and thrombin (T, 1 U/mL) positive controls. Hirudin reduced P-selectin expression (**A**, n=4) and microvesiculation (**B**, n=3-6) in response to RacL11 or thrombin. * p < 0.05 versus untreated PRP.

In RacL11-exposed washed ACD-anticoagulated platelets, P-selectin expression did not occur in the absence of plasma, but was still present in the thrombin-convulxin (TC, 0.15 U/mL-0.05 ug/mL) control. Addition of citrate-anticoagulated equine (E) or human (H) PDMP-depleted plasma (MDP) containing all coagulation factors re-established P-selectin expression in washed platelets (**C**, n=3). In contrast, microvesiculation was still induced by RacL11 in washed platelets without MDP, but addition of equine or human MDP boosted the percentage of PDMPs, with human MDP having a weaker response in virus-exposed platelets (**D**, n=6). * p <0.05 versus virus-exposed washed platelets with no added plasma.

Addition of FVII- or FX-deficient human MDP, instead of human MDP replete in all coagulation factors (Full), did not result in P-selectin expression in RacL11-exposed washed platelets. However, supplementation of FVII-deficient MDP with purified human FVIIa (1 nM, FVII- + FVIIa) re-established P-selectin expression induced by RacL11, indicating FX generation was secondary to extrinsic pathway activation (**E**, n=3 to 7). Microvesiculation was also significantly reduced in FVII- and FX-deficient MDP, with supplemental FVIIa boosting the response in FVII-deficient MDP (**F**, n=4). * p <0.05 versus Full MDP.

In contrast, addition of human FIX-, FXI- or FXII-deficient MDP to washed platelets did not significantly affect P selectin expression (**G**) or PDMP release (**H**) (n=3 to 7). Similar results were seen with Ab4 (S2 Fig).

**EHV-1-induced platelet activation is mediated by the tissue factor-factor VIIa complex and not viral glycoprotein C**

Using a combination of functional clotting assays with FVII- and FX-deficient plasma, inhibitory anti-TF antibodies and flow cytometry and electron microscopy techniques, Sutherland et al have shown that HSV envelopes constitutively express TF and phospholipid derived from the host cell [29]. We also found that virus alone binds Annexin V, suggesting the envelope expresses phosphatidylserine (Fig. 2). We performed a procoagulant assay to test if EHV-1 could activate FX in the absence of plasma or platelets. We found that both EHV-1 strains activated FX in a one-stage amidolytic assay in a PFU/cell-dependent manner, with RacL11 generating more FXa, consistent with its higher platelet agonist activity (Table 2). This response mimicked the induced platelet activation, since no FXa generation was seen with virus equivalent to 0.1 PFU/cell. Factor Xa was not generated without exogenous FVIIa, supporting a role for virus-associated TF in the procoagulant activity.

**Table 2. Equid herpesvirus type 1 generates factor Xa (FXa) in a dose-dependent manner.**

|  | Mean ± SD factor Xa (nM) | |
| --- | --- | --- |
| PFU/cell | RacL11 | Ab4 |
| 0.1 | 0* | 0* |
| 0.5 | 0.65 ± 0.12 | 0.21 ± 0.03 |
| 1.0 | 1.81 ± 0.20 | 0.54 ± 0.24 |

Factor Xa generation (nM) was measured with purified virus at different plaque forming units/cell (PFU/cell) using a one stage amidolytic assay with exogenous human FVIIa (1 nM) and FX (75 nM) and a chromogenic FXa substrate (n=4). The FXa concentration was derived from comparing the rate of substrate cleavage in the sample (Vmax) to a standard curve of serially diluted purified human FXa. Factor Xa generation was below the linearity of the assay (0.17 nM FXa) in negative controls (samples without added FVIIa or FX or RK lysate with all reagents). * Below assay linearity.

To more directly test the role of TF in thrombin generation and platelet activation, we pre-incubated a goat polyclonal anti-rabbit TF antibody (152 ug/mL) with an equivalent concentration of goat IgG as a negative control, with virus or RK lysate for 10-15 minutes at room temperature before adding the virus or controls to equine PRP. The anti-TF antibody abolished P-selectin expression on EHV-1-exposed platelets (Fig. 6A) and significantly inhibited microvesiculation (Fig. 6B), consistent with our results using washed platelets and human factor-deficient plasma. Although we used a goat polyclonal anti-TF antibody, the antibody was raised against rabbit TF, which is expressed in the RK cell line used to propagate the virus. This suggests that the virus-associated TF-dependent responses (FXa generation and platelet activation) were likely due to TF derived from the propagating host cell, as reported for HSV [29].

Previous studies with HSV have shown that the virus-encoded envelope protein gC is a weak direct activator of FX and co-operates with TF to generate FXa and promote virus replication within human umbilical vein endothelial cells [31,32]. We examined the role of gC in EHV-1-induced platelet activation by generating an Ab4-based gC deletion mutant (Fig. 1). The gC deletion mutant effectively activated platelets, inducing P-selectin expression and microvesiculation (Fig. 6C-D). We concluded that virus gC is not a major determinant of the observed effects of EHV-1 on platelet activation.

**Fig 6: Tissue factor mediates EHV-1-induced platelet activation, with no role for virus glycoprotein C.**

A goat polyclonal anti-rabbit tissue factor (TF) antibody (anti-TF, 152 ug/mL) abolished P-selectin expression (**A**) and significantly decreased the numbers of platelet-derived microparticles (PDMP) (**B**) in equine platelets exposed to RacL11 and Ab4 EHV-1 strains at 1 PFU/cell (n=6). Data shown is mean ± SD. * p < 0.001 for P-selectin and p = 0.025 for PDMPs versus goat IgG controls.

In contrast, platelets still expressed P-selectin (**C**) and shed PDMPs (**D**) when exposed to an Ab4-based envelope glycoprotein C deletion mutant (ΔgC) at 1 PFU/cell (n=8). Data shown is mean ± SD.

**EHV-1 associates with equine platelets**

To examine if EHV-1 binds to platelets, we exposed washed platelets (with or without 2.5 mM exogenous calcium) to EHV-1 for 10 minutes at 37°C, washed the platelets three times in PBS by centrifugation at 850 x *g* for 5 minutes at 22°C, then performed plaque assays on RK cells. Cells not exposed to virus and virus without cells treated to similar washes served as negative controls, whereas virus not subjected to the centrifugation washes was used as a positive control. We found that infectious virions could be retrieved from washed platelets (100 fold higher than free virus alone), suggesting that the virus binds to platelets without any requirement for plasma and calcium.

To determine if EHV-1 required platelets to generate thrombin or could generate thrombin in a plasma milieu alone, virus (at amounts equivalent to 1 PFU/cell) was incubated with equine CTI-MDP in the presence or absence of platelets and thrombin generation was measured using a fluorescent substrate. At 1 PFU/cell, the Ab4 strain generated small amounts of thrombin in CTI plasma alone without autologous platelets. Autologous platelets shortened the lag time and boosted thrombin generation with both virus strains (Fig. 7). Ab4 generated more thrombin than RacL11, despite having similar lag times with thrombin generation and lower procoagulant activity in the FXa-based amidolytic assay. This indicates that there are strain-dependent differences in amplification or propagation of thrombin generation on virus or platelet surfaces. No thrombin was generated with negative controls (CTI-MDP alone or with platelets, CTI-MDP with RK lysate with or without platelets).

**Fig. 7: EHV-1 generates thrombin in equine plasma.**

Thrombin generation by purified virus (at amounts equivalent to 1 PFU/cell) was measured using calibrated automated thrombography in corn trypsin inhibitor (CTI)-containing citrate-anticoagulated platelet-derived microparticle-depleted equine plasma (CTI-MDP) with or without autologous platelets (1 x 10^5^/reaction). Data shown is lag time (**A**), total thrombin generated (nM*minute, derived from the area under the thrombogram curve) (**B**) and compilation thrombogram curves (**C**, mean of 3 separate experiments). Addition of platelets shortened the lag time and boosted the total thrombin generated by both virus strains. Ab4, but not RacL11, generated small amounts of thrombin in CTI plasma alone. No thrombin was generated (lag time >30 minutes, total thrombin generated 0 nM*minute) in negative controls (CTI-MDP with or without autologous platelets and CTI-MDP with RK lysate with or without platelets).

Equine platelets express major histocompatibility complex class I molecules (MHC-I) [33], which is one of the entry receptors for the virus on other cells [25]. To test if MHC-I is a possible platelet receptor for the virus that mediates platelet activation, we exposed equine PRP to RacL11 in the presence of an inhibitory antibody against equine MHC-I (50 ug/mL, clone CZ3 [25], with isotype control). The anti-MHC-I antibody did not inhibit P-selectin expression or microvesiculation in EHV-1-exposed platelets, arguing against a role for MHC-I in virus-induced platelet responses.

**Discussion**

We show here for the first time that EHV-1 associates with and activates platelets, inducing α-granule secretion and microvesiculation. Platelet activation is mediated by thrombin generated through TF-FVIIa-triggered activation of FX. Our working model of EHV-1-induced platelet activation is that the virus rapidly binds to the outer platelet membrane. Once sufficient virus particles have attached (0.5 PFU/cell or higher), microvesiculation is induced and thrombin generation is triggered via TF incorporated within the virus envelope and propagated via virus- or platelet-associated phospholipid. This yields a platelet membrane-localized thrombin burst, which activates platelets via protease-activated receptors. Although free virus with both strains (depending on virus load) can generate thrombin in the absence of platelets, platelets amplify thrombin generation (Fig. 8). Our data suggests that enough viral particles are present at 0.5 PFU/cell (whether attached to platelets or free in plasma) to generate sufficient thrombin to elicit platelet activation of most platelets in the vicinity. Direct virus binding to platelets could potentially explain the concentration-dependent nature of the microvesiculation response.

**Fig. 8: Model for EHV-1-induced activation of platelets.**

In our model of EHV-1-induced platelet activation, we propose that the virus first binds to platelets. Concurrently, factor VII (FVII) forms a complex with TF in the viral envelope and activates factor X (FX), with FXa generating thrombin in the vicinity of or on the surface of platelets. Thrombin generation may be propagated by phosphatidylserine (PS) in the viral envelope. Thrombin binds to protease-activated receptors (PAR) on platelet surfaces, inducing release of α-granules with subsequent surface P-selectin and PS expression and microvesiculation. If marked, microvesiculation results in loss of membrane-anchored P-selectin on microparticles. Depending on the viral strain or load, thrombin may be generated on the surface of virus not attached to platelets.

Strain-dependent differences were observed in procoagulant activity, thrombin generation and platelet activation responses. The reason for these observed differences is unknown. It is possible that these differences are due to the inherent imprecision of plaque assays for virus quantification or a higher ratio of virions to PFU for RacL11 versus Ab4. However, the observed differential response was maintained across several virus preparations leading us to hypothesize that there are structural differences between the strains that affect their procoagulant ability. For instance, the higher procoagulant activity with RacL11 suggests this strain contains more TF in its envelope or TF is in a “decrypted”, more procoagulant conformational state. Surprisingly, the higher procoagulant activity with the purified system did not translate into more robust thrombin generation in a plasma milieu with RacL11, even though this strain induced stronger microvesiculation. This could be due to strain-related differences in envelope phospholipid content or glycoproteins, which may affect assembly or activity of coagulation factors on the virus or platelet surface. There are minor strain-associated differences in envelope glycoprotein sequences, but this does not translate into differences in virus attachment on other cells [34]. RacL11 has a partial or complete deletion in open reading frames (ORF) 1 and 2 at the extreme left terminus of its genome [35]. ORF1 and 2 encode early gene products and not envelope proteins, however ORF1, and possibly ORF2, do encode proteins that affect vesicle transport and protein expression and these mutations could account for the observed strain differences [36]. Unlike HSV, in which virus gC acts as a cofactor for virus TF-induced procoagulant activity [31,32], we found that gC was not required for EHV-1-induced platelet activation.

Higher infectious viral doses, particularly with the RacL11 strain, yielded strong microvesiculation. When marked, this microvesiculation was accompanied by a decrease in P-selectin expression, which was not seen with thrombin or thrombin-convulxin stimulation. This was attributed to loss of membrane-anchored P-selectin as a consequence of microvesiculation, as observed previously with calcium ionophore-treated equine platelets [27]. The loss of P-selectin was more apparent with the RacL11 strain versus the Ab4 strain, even at similar degrees of microvesiculation. We hypothesize that this also reflects strain-related differences in how the virus binds to or activates platelets, with RacL11 potentially inducing more robust calcium release, similar to that achieved by calcium ionophore. It is also possible that microvesiculation represents apoptosis and not activation per se, as recently described for Dengue virus [16]. We have found that higher infectious doses of EHV-1 (5 PFU/cell) perturb mitochondrial membrane permeability in monocytes, consistent with induction of apoptosis [22]. However, the rapidity of the EHV-1-induced response (minutes versus 6 hours for Dengue virus) argues against an apoptotic mechanism for platelet microvesiculation with EHV-1.

How the virus associates with platelets is unknown. Equine platelets express MHC-I [33], a known cellular receptor for virus entry [25], however we found that an inhibitory anti-MHC-I antibody did not block activation. It is known that the virus attaches to cells via envelope glycoproteins, particularly gC, which binds to heparin-like glycosaminoglycans on platelet surfaces, and that gD mediates interaction with MHC-I [34,37]. Our finding that the Ab4-based gC deletion mutant could still activate platelets suggests that gC is not required for binding, perhaps due to retention of gB or gD, which are also envelope attachment proteins. Alternatively, virus attachment could be mediated by host-derived phosphatidylserine within the virus envelope binding to platelet receptors, either directly or through bridging molecules such as Gas6 [38]. It is likely the virus acquires phosphatidylserine from host cell membranes, as described for HSV and human cytomegalovirus [29,39]. It is also possible that the virus attaches to integrins on platelet membranes, as described for HIV [40]. These findings indicate that viruses can bind to and activate platelets through various mechanisms, likely in a virus-specific manner. Further studies are needed to elucidate how EHV-1 associates with platelets and if it is internalized after binding. Unlike other species, equine platelets lack an open canalicular system [41], which may facilitate virus uptake, as described for pathogenic human viruses [10,11].

Virus-induced platelet activation can have several consequences for the host. Activation (or apoptosis) could be part of a host protective or innate immune response to virus infection and may facilitate viral clearance. This immune response can improve survival in virus-infected hosts, despite an ensuing thrombocytopenia that can manifest as hemorrhage if sufficiently severe [14,42,43]. Platelet counts decreased by an average of 50% between days 1-9 after experimental infection with Ab4 in 8 Welsh mares [18], which could be due to clearance of activated platelets. Platelets could also be a source of infectious virus for leukocytes and endothelial cells. Activated platelets bind to leukocytes and endothelial cells via P-selectin and PSGL-1 interactions [7,44], which we hypothesize could promote virus infection of endothelial cells and the subsequent vasculitis that is a pathological feature of EHV-1 infection [18-21]. Platelet-derived microparticles shed from activated platelets could also cause vascular injury and promote virus dissemination. Microparticles released from Dengue-exposed platelets *in* *vitro* or *in vivo* contain high concentrations of interleukin-1β (IL-1β) and increase vascular permeability in an IL-1β-dependent manner [45]. Potentially, PDMPs shed from virus-activated platelets could contribute to endothelial dysfunction in hemorrhagic viral infections, such as Dengue and Ebola virus. It is also possible that EHV-1-activated platelets could contribute directly to a hypercoagulable state, predisposing horses to the thrombosis that is observed in several vascular endothelial beds, including the respiratory system, placenta and neurologic tissue, during infection [18-20]. Finally, internalization and sequestration of virus within platelets could potentially help the virus evade the immune system and contribute to virus latency, a characteristic feature of EHV-1 [17].

In conclusion, our studies highlight a new mechanism by which an enveloped virus, EHV-1, can rapidly activate platelets, namely through generation of thrombin by virus-associated TF. The virus-associated TF is likely derived from the host cell membrane and expressed on the virus envelope. We hypothesize that other enveloped viruses, such as cytomegalovirus and HSV, which incorporate TF within their envelope [29], could potentially activate platelets in a similar manner to EHV-1. We speculate that virus-induced platelet activation could be part of the innate immune response, facilitating viral clearance, but could also potentially contribute to the pathologic sequelae associated with viral infections, such as thrombosis, inflammation, endothelial dysfunction and dissemination of infection.

**Acknowledgments**

The authors acknowledge Yoojin Park for assisting Nicole DeAngelis with the thrombin generation assay, Alexa Fland and other staff at the Equine Research Park for help with blood sampling of horses, and Dr. Marjory Brooks for critical review of the manuscript and advice on data interpretation.

**References**

1. McSorley J, Shapiro L, Brownstein MH, Hsu KC (1974) Herpes simplex and varicella-zoster: comparative histopathology of 77 cases. Int J Dermatol 13: 69-75.

2. Bunce PE, High SM, Nadjafi M, Stanley K, Liles WC, Christian MD (2011) Pandemic H1N1 influenza infection and vascular thrombosis. Clin Infect Dis 52: e14-17.

3. Geisbert TW, Young HA, Jahrling PB, Davis KJ, Kagan E, Paragas J, et al. (2003) Mechanisms underlying coagulation abnormalities in ebola hemorrhagic fever: overexpression of tissue factor in primate monocytes/macrophages is a key event. J Infect Dis 188: 1618-1629.

4. Vercellotti GM (1990) Proinflammatory and procoagulant effects of herpes simplex infection on human endothelium. Blood Cells 16: 209-215; discussion 215-206.

5. de Witt SM, Verdoold R, Cosemans JM, Heemskerk JW (2014) Insights into platelet-based control of coagulation. Thromb Res 133 Suppl 2: S139-148.

6. Sinauridze EI, Kireev DA, Popenko NY, Pichugin AV, Panteleev MA, Krymskaya OV, et al. (2007) Platelet microparticle membranes have 50- to 100-fold higher specific procoagulant activity than activated platelets. Thromb Haemost 97: 425-434.

7. Yang J, Furie BC, Furie B (1999) The biology of P-selectin glycoprotein ligand-1: its role as a selectin counterreceptor in leukocyte-endothelial and leukocyte-platelet interaction. Thromb Haemost 81: 1-7.

8. Slotta JE, Braun OO, Menger MD, Thorlacius H (2009) Capture of platelets to the endothelium of the femoral vein is mediated by CD62P and CD162. Platelets 20: 505-512.

9. Oostingh GJ, Pozgajova M, Ludwig RJ, Krahn T, Boehncke WH, Nieswandt B, et al. (2007) Diminished thrombus formation and alleviation of myocardial infarction and reperfusion injury through antibody- or small-molecule-mediated inhibition of selectin-dependent platelet functions. Haematologica 92: 502-512.

10. Agbanyo FR, Wasi S (1994) Human cytomegalovirus interaction with platelets and adhesive glycoproteins: significance in viral pathogenesis. J Infect Dis 170: 1120-1127.

11. Pugliese A, Gennero L, Cutufia M, Enrietto M, Morra E, Pescarmona P, et al. (2004) HCV infective virions can be carried by human platelets. Cell Biochem Funct 22: 353-358.

12. Youssefian T, Drouin A, Masse JM, Guichard J, Cramer EM (2002) Host defense role of platelets: engulfment of HIV and Staphylococcus aureus occurs in a specific subcellular compartment and is enhanced by platelet activation. Blood 99: 4021-4029.

13. Noisakran S, Gibbons RV, Songprakhon P, Jairungsri A, Ajariyakhajorn C, Nisalak A, et al. (2009) Detection of dengue virus in platelets isolated from dengue patients. Southeast Asian J Trop Med Public Health 40: 253-262.

14. Koupenova M, Vitseva O, MacKay CR, Beaulieu LM, Benjamin EJ, Mick E, et al. (2014) Platelet-TLR7 mediates host survival and platelet count during viral infection in the absence of platelet-dependent thrombosis. Blood 124: 791-802.

15. Mayne E, Funderburg NT, Sieg SF, Asaad R, Kalinowska M, Rodriguez B, et al. (2012) Increased platelet and microparticle activation in HIV infection: upregulation of P-selectin and tissue factor expression. J Acquir Immune Defic Syndr 59: 340-346.

16. Hottz ED, Oliveira MF, Nunes PC, Nogueira RM, Valls-de-Souza R, Da Poian AT, et al. (2013) Dengue induces platelet activation, mitochondrial dysfunction and cell death through mechanisms that involve DC-SIGN and caspases. J Thromb Haemost 11: 951-962.

17. Lunn DP, Davis-Poynter N, Flaminio MJ, Horohov DW, Osterrieder K, Pusteria N, et al. (2009) Equine herpesvirus-1 consensus statement. J Vet Intern Med 23: 450-461.

18. Edington N, Bridges CG, Patel JR (1986) Endothelial cell infection and thrombosis in paralysis caused by equid herpesvirus-1: equine stroke. Arch Virol 90: 111-124.

19. Smith KC, Mumford JA, Lakhani K (1996) A comparison of equid herpesvirus-1 (EHV-1) vascular lesions in the early versus late pregnant equine uterus. J Comp Pathol 114: 231-247.

20. Smith KC, Whitwell KE, Mumford JA, Gower SM, Hannant D, Tearle JP (1993) An immunohistological study of the uterus of mares following experimental infection by equid herpesvirus 1. Equine Vet J 25: 36-40.

21. Szeredi L, Aupperle H, Steiger K (2003) Detection of equine herpesvirus-1 in the fetal membranes of aborted equine fetuses by immunohistochemical and in-situ hybridization techniques. J Comp Pathol 129: 147-153.

22. Yeo WM, Osterrieder N, Stokol T (2013) Equine herpesvirus type 1 infection induces procoagulant activity in equine monocytes. Vet Res 44: 16.

23. Reczko E, Mayr A (1063) The ultrastructure of a virus of the herpes group isolated from a horse. Arch Ges Virusforsch 13: 591-593.

24. Tischer BK, von Einem J, Kaufer B, Osterrieder N (2006) Two-step red-mediated recombination for versatile high-efficiency markerless DNA manipulation in Escherichia coli. Biotechniques 40: 191-197.

25. Kurtz BM, Singletary LB, Kelly SD, Frampton AR, Jr. (2010) Equus caballus major histocompatibility complex class I is an entry receptor for equine herpesvirus type 1. J Virol 84: 9027-9034.

26. Shah MD, Bergeron AL, Dong JF, Lopez JA (2008) Flow cytometric measurement of microparticles: pitfalls and protocol modifications. Platelets 19: 365-372.

27. Brooks MB, Divers T, Watts AE, Ness S, Frye A, Stokol T, et al. (2013) A Randomized Study of the Effects of Clopidogrel on Parameters of Equine Platelet Activation Response Am J Vet Res 74: 1212-1222.

28. Segura D, Monreal L, Espada Y, Pastor J, Mayos I, Homedes J (2005) Assessment of a platelet function analyser in horses: reference range and influence of a platelet aggregation inhibitor. Vet J 170: 108-112.

29. Sutherland MR, Raynor CM, Leenknegt H, Wright JF, Pryzdial EL (1997) Coagulation initiated on herpesviruses. Proc Natl Acad Sci U S A 94: 13510-13514.

30. Gershom ES, Sutherland MR, Lollar P, Pryzdial EL (2010) Involvement of the contact phase and intrinsic pathway in herpes simplex virus-initiated plasma coagulation. J Thromb Haemost 8: 1037-1043.

31. Sutherland MR, Friedman HM, Pryzdial EL (2004) Herpes simplex virus type 1-encoded glycoprotein C enhances coagulation factor VIIa activity on the virus. Thromb Haemost 92: 947-955.

32. Sutherland MR, Ruf W, Pryzdial EL (2012) Tissue factor and glycoprotein C on herpes simplex virus type 1 are protease-activated receptor 2 cofactors that enhance infection. Blood 119: 3638-3645.

33. Hirni H, Lazary S, Gerber H (1988) Serological and genetic study of the ELA W21 specificity. Anim Genet 19: 391-394.

34. Osterrieder N (1999) Construction and characterization of an equine herpesvirus 1 glycoprotein C negative mutant. Virus Res 59: 165-177.

35. Kim SK, Jang HK, Albrecht RA, Derbigny WA, Zhang Y, O'Callaghan DJ (2003) Interaction of the equine herpesvirus 1 EICP0 protein with the immediate-early (IE) protein, TFIIB, and TBP may mediate the antagonism between the IE and EICP0 proteins. J Virol 77: 2675-2685.

36. Ma G, Feineis S, Osterrieder N, Van de Walle GR (2012) Identification and characterization of equine herpesvirus type 1 pUL56 and its role in virus-induced downregulation of major histocompatibility complex class I. J Virol 86: 3554-3563.

37. Akhtar J, Shukla D (2009) Viral entry mechanisms: cellular and viral mediators of herpes simplex virus entry. FEBS J 276: 7228-7236.

38. Morizono K, Chen IS (2014) Role of phosphatidylserine receptors in enveloped virus infection. J Virol 88: 4275-4290.

39. Pryzdial EL, Wright JF (1994) Prothrombinase assembly on an enveloped virus: evidence that the cytomegalovirus surface contains procoagulant phospholipid. Blood 84: 3749-3757.

40. Wang J, Zhang W, Nardi MA, Li Z (2011) HIV-1 Tat-induced platelet activation and release of CD154 contribute to HIV-1-associated autoimmune thrombocytopenia. J Thromb Haemost 9: 562-573.

41. Choi W, Karim ZA, Whiteheart SW (2010) Protein expression in platelets from six species that differ in their open canalicular system. Platelets 21: 167-175.

42. Othman M, Labelle A, Mazzetti I, Elbatarny HS, Lillicrap D (2007) Adenovirus-induced thrombocytopenia: the role of von Willebrand factor and P-selectin in mediating accelerated platelet clearance. Blood 109: 2832-2839.

43. Alonzo MT, Lacuesta TL, Dimaano EM, Kurosu T, Suarez LA, Mapua CA, et al. (2012) Platelet apoptosis and apoptotic platelet clearance by macrophages in secondary dengue virus infections. J Infect Dis 205: 1321-1329.

44. Passacquale G, Vamadevan P, Pereira L, Hamid C, Corrigall V, Ferro A (2011) Monocyte-platelet interaction induces a pro-inflammatory phenotype in circulating monocytes. PLoS One 6: e25595.

45. Hottz ED, Lopes JF, Freitas C, Valls-de-Souza R, Oliveira MF, Bozza MT, et al. (2013) Platelets mediate increased endothelium permeability in dengue through NLRP3-inflammasome activation. Blood 122: 3405-3414.

**Supplemental information legends**

**S1 Fig. EHV-1 induces platelet activation in equine platelet-rich plasma in a concentration-dependent manner.**

Platelets were exposed for 10 minutes to RacL11 and Ab4 EHV-1 strains at increasing PFU/cell (0.01, 0.1, 0.5, 1 or 5) with rabbit kidney (RK) lysate as a negative control. The mean ± SD percentage of platelets positive for P-selectin (**A**) or PDMPs (**B**) was then quantified (n=4). At the higher PFUs/cell of 0.5, 1 and 5, both strains induced P-selectin expression and microvesiculation. RacL11 induced slightly stronger microvesiculation than Ab4, with a decrease in P-selectin expression at the higher PFU/cell of 1 and 5.

* p < 0.001 for P-selectin and p = 0.002 for PDMP versus RK, 0.01 and 0.1 PFU/cell for each virus strain (except Ab4 at 0.5 PFU/cell was not significantly different from RK). ** p < 0.001 versus Ab4.

**S2 Fig. The Ab4 strain of EHV-1 induces platelet activation through factor VII-generated thrombin.**

Addition of hirudin (10 units) to equine citrate-anticoagulated platelet-rich plasma reduced P-selectin expression (**A**) and release of platelet-derived microparticles (PDMPs; **B**) in response to Ab4 at 1 plaque forming unit/cell or thrombin (T, 1 U/mL) (n=3-5). No P-selectin expression or PDMP release occurred in PBS-treated negative control platelets. * p < 0.05 versus untreated platelets.

P-selectin expression was abolished in washed platelets exposed to Ab4, but re-established with addition of platelet-derived microparticle-depleted citrate-anticoagulated equine (E) or human plasma containing all coagulation factors (Full) or human plasma deficient in factors IX, XI or XII. In contrast, addition of human FVII- or FX-deficient plasma did not re-establish P-selectin expression, unless supplemental purified FVIIa (1 nM) was added to FVII-deficient plasma (FVII- + FVIIa) (**C**, n=4). * p < 0.05 versus washed platelets with no added plasma, ** p < 0.05 versus Full plasma.

The release of PDMPs was boosted in virus-exposed washed platelets in the presence of equine or human plasma. The degree of microvesiculation was significantly decreased when FVII- or FX-deficient plasma was added to Ab4-exposed washed platelets and supplemental purified human FVIIa significantly boosted PDMP percentages in FVII-deficient plasma (**D**, n=3). Data shown are mean ± SD. * p < 0.05 versus washed platelets with no added plasma, ** p < 0.05 versus Full plasma.

**S3 Fig. Equid herpesvirus type 1 (EHV-1)-induced platelet activation is not affected by corn trypsin inhibitor (CTI).**

Platelet-rich plasma prepared from blood collected into citrate anticoagulant with or without CTI (50 ug/mL) was exposed to the RacL11 and Ab4 strains of EHV-1 at 1 plaque forming unit/cell or rabbit kidney (RK) cell lysate for 10 minutes at 37°C, then the mean ± SD percentage of platelets expressing P-selectin (**A**) or platelet-derived microparticles (PDMPs, **B**) was quantified by flow cytometry (n=3). CTI did not significantly inhibit these markers of platelet activation
